# Online Adaptation to Altered Auditory Feedback Is Predicted by Auditory Acuity and Not by Domain-General Executive Control Resources

**DOI:** 10.3389/fnhum.2018.00091

**Published:** 2018-03-12

**Authors:** Clara D. Martin, Caroline A. Niziolek, Jon A. Duñabeitia, Alejandro Perez, Doris Hernandez, Manuel Carreiras, John F. Houde

**Affiliations:** ^1^Basque Center on Cognition, Brain and Language, San Sebastian, Spain; ^2^IKERBASQUE, Basque Foundation for Science, Bilbao, Spain; ^3^Department of Communication Sciences and Disorders, University of Wisconsin–Madison, Madison, WI, United States; ^4^Facultad de Lenguas y Educación, Universidad Nebrija, Madrid, Spain; ^5^Department of Psychology, Center for Interdisciplinary Brain Research, University of Jyväskylä, Jyväskylä, Finland; ^6^Basque Language and Communication Department, University of the Basque Country, San Sebastian, Spain; ^7^Department of Otolaryngology, University of California, San Francisco, San Francisco, CA, United States

**Keywords:** speech production, altered feedback, adaptation, auditory acuity, executive control

## Abstract

When a speaker's auditory feedback is altered, he adapts for the perturbation by altering his own production, which demonstrates the role of auditory feedback in speech motor control. In the present study, we explored the role of auditory acuity and executive control in this process. Based on the DIVA model and the major cognitive control models, we expected that higher auditory acuity, and better executive control skills would predict larger adaptation to the alteration. Thirty-six Spanish native speakers performed an altered auditory feedback experiment, executive control (numerical Stroop, Simon and Flanker) tasks, and auditory acuity tasks (loudness, pitch, and melody pattern discrimination). In the altered feedback experiment, participants had to produce the pseudoword “pep” (/pep/) while perceiving their auditory feedback in real time through earphones. The auditory feedback was first unaltered and then progressively altered in F1 and F2 dimensions until maximal alteration (F1 −150 Hz; F2 +300 Hz). The normalized distance of maximal adaptation ranged from 4 to 137 Hz (median of 75 ± 36). The different measures of auditory acuity were significant predictors of adaptation, while individual measures of cognitive function skills (obtained from the executive control tasks) were not. Better auditory discriminators adapted more to the alteration. We conclude that adaptation to altered auditory feedback is very well-predicted by general auditory acuity, as suggested by the DIVA model. In line with the framework of motor-control models, no specific claim on the implication of executive resources in speech motor control can be made.

## Introduction

Sensorimotor control has been studied for decades by exploring the role of visual feedback in reaching. Many studies have shown that when participants reach for a target, they initially miss it when the visual feedback of their hand position is shifted. This visual feedback alteration, when it is consistent, induces participants to adapt, and adjust their reaches to oppose the feedback shift. This learned adaptation, developed gradually in response to consistently altered feedback, is called sensorimotor adaptation (von Helmholtz, 1962; Welch, 1978; Rossetti et al., 1993; Redding et al., 2005). Drawing a parallel between visual and auditory feedback, several authors have shown that speakers adapt for alteration of auditory feedback during speech production. Both phenomena are explained by the existence of an internal forward model that enables human subjects to adjust their motor act on-line to any perturbation (Houde and Jordan, 1998, 2002; Purcell and Munhall, 2006a). This sensorimotor adaptation (SA) is highly variable across individuals. The goal of the present study is to identify some critical factors involved in SA using a speech production task.

### Sensorimotor adaptation in speech

It has long been known that auditory feedback has an impact on speech: speakers respond to an increase in surrounding noise level by concomitantly increasing the volume of their speech (Lombard, 1911; Lane and Tranel, 1971), and disruptions of speech are consistently observed when auditory feedback is delayed (Lee, 1950; Yates, 1963). Speakers have also been shown to adapt for unpredictable shifts in pitch (Elman, 1981; Burnett et al., 1998), loudness (Heinks-Maldonado and Houde, 2005; Bauer et al., 2006), and formant frequencies (Purcell and Munhall, 2006a; Tourville et al., 2008; Cai et al., 2012; Niziolek and Guenther, 2013). In addition to these rapid adaptation responses to online feedback shifts, SA in vowel production was observed when speakers listened to their auditory feedback altered by a consistent, learnable shift in formants (Houde and Jordan, 1998, 2002; Purcell and Munhall, 2006b; Villacorta et al., 2007), or fundamental frequency (Jones and Munhall, 2000, 2005; Xu et al., 2004), showing an aftereffect which persists after the removal of altered feedback and which is thus separable from online adaptation.

Sensorimotor adaptation can be explained in the following way: the perceived feedback during the execution of an action is compared with predicted feedback, and any inconsistency between the actual and predicted feedback lead to changes (adaptation) in the motor command parameters (Held and Hein, 1958; Hein and Held, 1962; Welch, 1986). The predicted feedback is generated internally from the conjunction of an efference copy of the motor command parameters with an internal model of both the motor system and the environment (“forward models,” see Jordan and Rumelhart, 1992; Guenther, 1995; Wolpert et al., 1995; Perkell et al., 1997; Tremblay et al., 2003; e.g., DIVA model, Guenther et al., 1998, 2006; Tourville and Guenther, 2011; State Feedback Control model of speech motor control, Houde and Nagarajan, 2011).

Regarding the role of auditory feedback in speech production, the forward model would function as follows: When a speaker produces a speech sound, the motor command parameters are sent to the articulatory system while an efference copy of the descending motor command parameters is created. Based on the efference copy, the forward model generates a prediction of what should be the feedback of this oral production. Then, the actual sensory feedback perceived during speech sound production is compared to the predicted feedback. Whenever there is a mismatch between the actual and predicted feedback, there is *online adaptation*. For example, when a formant alteration is artificially introduced in the actual feedback, a mismatch will be detected, and adaptive motor command parameters will result in the production of a shifted version of the speech sound (Houde and Jordan, 1998, 2002; Purcell and Munhall, 2006a). The online change in motor commands after repeated mismatches leads to a long-lasting change in the motor command parameters. In fact, once the alteration in auditory feedback is removed, there is persistence in producing the shifted version of the speech sound, as shown by the adaptation *aftereffect* (Houde and Jordan, 1998, 2002; Purcell and Munhall, 2006a). Thus, while *adaptation* reflects online changes in motor commands, *aftereffects* can be attributed to persistent recalibration of the target sound which washes out when the alteration is removed.

### Variability in adaptation and aftereffect

*Adaptation* and *aftereffect* are usually explored through formant alteration in auditory feedback during syllable production. Participants have to pronounce a syllable each time a cue appears on a computer screen. They listen to their auditory feedback through earphones in real time. Formants are not shifted during the initial trials (baseline trials). Then, the first and/or second vowel formant frequencies (F1 and/or F2) are progressively shifted during “ramp” trials to reach a maximal alteration of F1 ± x Hz / F2 ± y Hz. The participant is typically unaware of the alteration and continues to produce the same syllable while unconsciously modulating the verbal production as a consequence of the external alteration (hold trials). The *adaptation* to alteration is measured as the difference in vowel formants produced during hold vs. baseline trials. At the end of the experiment, the alteration in feedback is removed for the last block of trials. The *aftereffect* to alteration is measured as the difference in vowel formants produced during end vs. baseline trials. Several studies using similar designs consistently report significant *adaptation* and *aftereffect* at the group level: Participants generally alter their production so as to oppose the shift of the feedback alteration (adaptation) and this alteration of production is maintained even after removing the alteration in feedback (aftereffect; Houde and Jordan, 1998, 2002; Purcell and Munhall, 2006a). Nevertheless, when looking at individual adaptation and aftereffect values, a large variability is observed. For instance, Purcell and Munhall (2006b), despite the main significant adaptation, report a large variability in individual adaptation (from 9 to 126 Hz for a 200 Hz alteration). Houde and Jordan (2002) report that some participants adapt almost completely while others do not show any significant adaptation (see Figures 5A,C in Houde and Jordan, 2002; Figure 3 in Houde and Jordan, 1998; see also Villacorta et al., 2007). Cai et al. (2010) observed a significant adaptation to F1 alteration in only 60% of their participants. Note that large individual variability in adaptation and aftereffect was also reported in pitch-shift studies (e.g., Burnett et al., 1998; Sivasankar et al., 2005).

One tentative explanation of variability in adaptation and aftereffect can be found in the literature on executive control. According to the major theoretical accounts of cognitive control (Botvinick et al., 2001; Holroyd et al., 2005), performance is adjusted when a conflict is encountered, and this “conflict monitoring” skill varies among people. During speech production, two sources of information have to be integrated: top-down feedforward information and bottom-up auditory feedback (mandatorily but not exclusively, since other sources of information have to be integrated such as somatosensory information for instance; Lametti et al., 2012). When an alteration is applied to the bottom-up auditory feedback, a conflict between those two sources of information is introduced. If such conflict monitoring requires executive functioning, one would expect people with lowest executive control skills to be poor in adaptation (i.e., poor in conflict monitoring and thus poor in resolving conflict between the two sources of information).

Secondly, according to some theoretical postulates (e.g., DIVA model), auditory acuity should be another factor explaining variability in adaptation and aftereffect (Guenther et al., 1998, 2006; Tourville and Guenther, 2011). In the model, speakers' targets for production are defined as sensory goals in a state space, and more acute speakers have smaller target regions. This assumption is supported by previous work showing that more acute speakers have reduced production variability (Perkell et al., 2004, 2008; Franken et al., 2017) and by models that equate acuity with higher resolution in auditory space, leading to smaller (more precise) target regions (Perkell, 2007, 2012). Here, we can infer that the same feedback alteration will push productions farther outside the target region for individuals with higher auditory acuity (i.e., with smaller target regions). Thus, higher auditory acuity should lead to finer detection of subtle alterations (errors) in auditory feedback, leading to larger amount of adaptation.

In the present study, we investigated the relationship between adaptation and aftereffect and various factors that could play a relevant role in SA. In line with cognitive control models and the DIVA model, we specifically targeted two main factors: executive control and auditory acuity. First, if good adapters are better in conflict detection/monitoring they should outperform poor adapters in executive control. Second, if good adapters are better in detecting subtle alterations in feedback perception (i.e., smaller target regions) they should outperform poor adapters in auditory acuity. In other words, we tested the hypotheses that poor adapters are less sensitive to feedback alterations because (1) they perceive their feedback but they do not efficiently monitor errors in it (variability in adaptation would then be mainly explained by executive control skills) or because (2) they cannot perceive their feedback as accurately (variability in adaptation would then be mainly explained by auditory acuity). Here we explore these two hypotheses in greater depth.

### SA and executive control

Since one possible explanation for variability in adaptation and aftereffect is differing efficiency of conflict monitoring, we investigated the relationship between executive control and adaptation/aftereffect to altered feedback. The underlying hypothesis is that the same networks that mediate domain-general conflict monitoring (cognitive control) also allow better internal self-speech monitoring (Schiffer et al., 2015). We hypothesized that the better the participants are in detecting, monitoring, and resolving conflict between two sources of information, the more they adapt to altered feedback. Such interplay between domain-general and speech-specific monitoring is still debated, and previous literature reveals contradictory results on this topic.

On one hand, control of auditory feedback during self-produced speech perception is thought to be involuntary in nature, independent of general cognitive executive resources (see Hu et al., 2015). In fact, several studies have shown that participants were unable to voluntarily suppress the adaptive response induced by F0 alteration in their feedback (Munhall et al., 2009; Keough et al., 2013; Patel et al., 2014), even when explicitly instructed to ignore the altered feedback (Zarate and Zatorre, 2008; Hu et al., 2015). Moreover, internal forward models do not make claims about general executive resources in auditory feedback processing (e.g., Hickok et al., 2011; Houde and Nagarajan, 2011). On the other hand, recent studies revealed that feedback control can be modulated by experimental task (e.g., speaking vs. singing; Natke et al., 2003), learning experience (tone language experience; Chen et al., 2012; singing experience; Zarate and Zatorre, 2008), and auditory attentional load (Tumber et al., 2014). Zarate and Zatorre (2008), for instance, showed that adaptive responses to altered feedback can be suppressed when subjects are instructed to ignore their feedback, but this inhibition capacity was observed only in musicians. Tumber et al. (2014) showed that when less attention was available for auditory feedback monitoring (dual-task condition producing a high attentional load) participants adapted less to pitch alterations in auditory feedback (see also Liu et al., 2015 and Scheerer et al., 2016, for evidence of the influence of attention on adaptation).

Thus, recent studies suggest that the level of reliance on auditory feedback during self-produced speech listening can vary, even if involuntary in nature. Because auditory attentional load modulates responses to altered auditory feedback (Tumber et al., 2014; Liu et al., 2015; Scheerer et al., 2016), it may be hypothesized that executive control resources play a role in processing competing feedforward and feedback sources of information. Here, we go a step further on the implication of general executive skills in altered feedback processing. We make the hypothesis that the level of adaptation to altered feedback also depends on general executive control skills. We test the prediction that the higher the general executive control skills, the higher the capacity of detecting and monitoring conflict, and consequently the higher the adaptation to the altered feedback.

In order to investigate the role of executive control in altered auditory feedback processing, we measured the correlation between general executive control skills, and adaptation to altered auditory feedback. Participants had to perform a CVC production task in which auditory feedback was progressively altered. In a second experimental session, we independently estimated the executive control skills of each participant. In order to estimate conflict monitoring skills, we used classical executive control tasks including confliction resolution (Simon task, Simon and Rudell, 1967; Flanker task, Eriksen and Eriksen, 1974; numerical Stroop task, Tzelgov et al., 1992). Resolution of conflict between two sources of information is assessed by interference effects in those tasks: the larger the cognitive control skills, the smaller the interference effect (see Methods section for further details). Those tasks are the most commonly used in the literature to measure domain-general executive control capacities (using non-verbal stimuli) in a modality-independent manner (Roberts et al., 2006; Spagna et al., 2015). Three different tasks targeting executive control have been included since they measure different sub-types of conflict monitoring related to various forms of inhibitory processes (Miyake and Friedman, 2012; Duñabeitia et al., 2014). If domain-general executive control skills influence altered auditory feedback processing, we should expect those with the strongest executive control skills to exhibit the largest adaptation to altered feedback. If, as suggested by internal forward models, general executive control resources do not play a relevant role in auditory feedback processing (e.g., Hickok et al., 2011; Houde and Nagarajan, 2011), no correlation between executive control skills and adaptation should be observed.

### SA and auditory acuity

One of the main predictions of the DIVA model is that auditory perception affects development of speech motor commands, so that better auditory acuity goes hand-in-hand with better-tuned speech production. In fact, the DIVA model learns the targets for speech sounds by taking an acoustic signal as input, which it uses to learn which motor commands match that signal. Having a more accurate representation of that input (i.e., higher auditory acuity) would result in a smaller region of acoustic space that produces a “match” with the target. Therefore, speech productions at the periphery of the target region would be recognized as an error (i.e., “mismatch”) only for the individuals with high enough acuity to distinguish the peripheral production from the target signal (see Figure 5 in Perkell, 2007)^1^. In other words, when auditory feedback is altered, the speaker adapts until formant values of her auditory feedback move into the target region. The extent of the target region being smaller for acuity speakers (Perkell, 2007, 2012), those speakers should better adapt their speech production to altered auditory feedback.

This prediction (greater adaptation to alteration for better auditory acuity) was explored by Villacorta et al. (2007). In this previous study, participants were exposed to a classical altered feedback experiment. Their feedback was altered online (F1 shift) and the authors observed significant adaptation (online effect during alteration). The authors also measured auditory acuity to vowel formant differences, and showed that auditory acuity significantly correlated with adaptation: the better the auditory acuity, the greater the adaptation to feedback alteration (Villacorta et al., 2007). Note, however, that the link between adaptation and auditory acuity still has to be explored since several further studies did not observe any evidence for cross-participant correlations between adaptation and auditory acuity for F1 (Feng et al., 2011; Cai et al., 2012). Furthermore, the evidence is scarce regarding the link between auditory acuity and aftereffect (when alteration removed). In a study comparing singers and non-singers, Jones and Keough (2008) observed that both groups adapted for F0 altered feedback, but only the singer group showed significant aftereffect. The results of this study suggest that auditory acuity (presumed to be larger in singers than non-singers) might affect aftereffects of adaptation more than adaptation itself.

In the present study, participants were tested in an altered auditory feedback paradigm similar to the one used by Villacorta et al. (2007), except that F1 and F2 were simultaneously shifted. We explored the correlation between adaptation and/or aftereffect and auditory acuity at the individual level. To extend previous results obtained by Villacorta et al. (2007), we estimated auditory acuity in several dimensions by using a series of four auditory tests: pitch, loudness, melody, and transposed melody discrimination. First, to extend previous results to general (and not speech-related) auditory acuity, we included tones instead of speech sounds in the tasks. Basic match/mismatch detection at low levels of acoustic processing was assessed through pitch and loudness discrimination tasks. Additionally, two melody discrimination tasks assessed participants' ability to detect changes in an incoming melody relative to a remembered target melody. We hypothesized that adaptation to altered feedback correlates with performance on these tasks, which parallel the comparison of incoming speech feedback with an internal representation of target speech sounds. Thus, we expected poor adapters to suffer from poor auditory acuity and vice-versa, for the 4 different auditory acuity sub-components tested here.

## Materials and methods

### Participants

Thirty-six native speakers of Spanish (18 females) took part in the experiment. All subjects were right handed, had normal, or corrected to normal vision and self-reported normal audition. They had no previous history of psychological or neurological disorders. Five participants were removed from analyses because of a large number of failed formant tracks (more than 50% of the trials; see below for explanation). Analyses were then performed on 31 participants. Their mean age was 23.8 ± 4.2 years old (range: 19–39). Despite the small sample size usually used in altered feedback experiments (around 10 participants; see for instance Houde and Jordan, 1998, 2002; Purcell and Munhall, 2006b; Villacorta et al., 2007), we tested three times more participants in order to achieve reasonable power in regression analyses. All participants were naïve to the purpose of the study. Participants received a payment of 10€ per h for their collaboration. This study was carried out in accordance with the recommendations of the BCBL ethics committee with written informed consent from all subjects. All subjects gave written informed consent in accordance with the Declaration of Helsinki. The protocol was approved by the BCBL ethics committee.

### Altered auditory feedback paradigm

The adaptation paradigm used a feedback alteration device (FAD) that was designed by the last author using digital speech processing methods. The FAD induced real-time alterations in the subject's own voice during vocalization. Participants were seated in front of a PC video monitor wearing a head-mounted microphone and Sennheiser Koss earphones. They were instructed to pronounce a bilabial consonant-vowel-consonant (CVC) non-word (/pep/). Speech was transduced by the microphone through a Delta 44 sound card and into a computer. Speech was analyzed and re-synthesized in real time by the FAD. The FAD implemented a formant-shifting acoustic transformation and returned the altered feedback via earphones (in place of normal auditory feedback) with a delay of 30 ms (below the 50 ms threshold where speakers begin to be noticeably affected by feedback delays; Kalinowski and Stuart, 1996). It also recorded the formants of each participant's utterance.

Within the FAD, an analysis-synthesis process repeatedly captured from the microphone 3 ms frames of the participant's speech (32 time samples at an 11.025 kHz sampling rate). Each frame was shifted into a 400 sample buffer, which was analyzed by computing a narrow-band magnitude frequency spectrum. Formants were isolated from the spectrum, modified, and recombined together with pitch and temporal envelope to create a new narrow-band magnitude spectrum. This way, frames were analyzed, modified and re-synthesized into new frames making up the altered speech output (for further details on the signal-processing device, see Katseff et al., 2012). Note that the formant tracking approach was based on linear prediction coding (LPC) analysis. LPC has proven to be a very successful approach to formant tracking for a majority of speakers and speech sounds, but there are always speakers for whom LPC analysis proves to be inaccurate and unstable (five among 36 participants in the present study). LPC analysis is generally good for voiced non-nasal vowels, but there are always a certain number of subjects whose productions will deviate from ideal “one tube” articulations, possibly nasalizing their productions or positioning their tongue so that side paths for air in the vocal tract are introduced. In such case, LPC analysis would be inaccurate resulting in a large amount of failed formant tracks. Furthermore, note that shifting formant peaks was accomplished by shifting poles of the LPC analysis of the input speech, and such pole movement (especially the F1 pole) would noticeably change the overall spectral amplitude if it was not adapted for in the output speech.

#### Experimental task

Participants were asked to pronounce the non-word “PEP” (/pep/) 100 times, once per trial, each time a pink triangle appeared on the computer screen (with a break after every 15 trials). This non-word was chosen because the /e/ target vowel was centered in the vocal space, allowing alteration toward an existing vowel (/i/), and adaptation also toward an existing vowel (/a/). The consonant /p/ was chosen because it is labial, which creates less interfering coarticulation (Recasens, 1999).

Participants were unaware that there were four phases in the experiment: (1) Baseline: No alteration was applied to trials 1–20. (2) Ramp: Trials 21–40 were progressively and linearly altered until maximal alteration (from vowel /e/ toward /i/). Progressively altering formant frequencies from /e/ toward /i/ involved decreasing F1 frequency and increasing F2 frequency (i.e., decreasing/increasing peaks of spectral power). For F1, the magnitude values of spectral power progressively decreased until a minimum of −150 Hz in 7.5 Hz steps. For F2, the spectral power increased in 15 Hz step sizes until a maximum of 300 Hz. Alteration was gradually introduced to minimize the participant's awareness of the alteration. (3) Hold: Trials 41–80 were kept maximally altered. (4) End: Finally, alteration was completely removed for productions 81 until 100. Participants' utterances were recorded and formants were tracked.

During baseline trials, when the participant produced the vowel /e/, his auditory feedback was of this same vowel sound /e/. During hold trials, if the participant produced the vowel /e/ with no adaptation, his auditory feedback would be close to /i/. By shifting the formants of his production toward /a/, a participant could shift his auditory feedback closer to the formants of his original unaltered auditory feedback of vowel sound /e/. It is worth mentioning that in the present experiment as in previous ones using the same paradigm, participants were unaware of the manipulation and did not consciously detect the feedback alteration during ramp and hold trials. They detected the sudden change back to baseline, but none of them realized that this change restored unaltered feedback.

#### Auditory altered feedback data processing

F1/F2 formant values of each participant's utterance (trial) were measured online by the apparatus, and used for data analyses. Formant values for each utterance were measured as the mean formant frequencies over the vowel interval of the utterance. The vowel interval was determined by thresholding the amplitude envelope of the utterance. The first 5 (baseline) trials were discarded from analyses as considered a period of signal equilibration (Purcell and Munhall, 2006b; Villacorta et al., 2007). Then, missed trial values (until four consecutive) due to a program failure in formant tracking, were created by interpolation of boundary data (Code available at http://www.mathworks.com/matlabcentral/fileexchange/loadFile.do?objectId=4551&objectType=file). This procedure was applied to 0.9 ± 1.4% of the trials on average (range of failed formant tracking from 0 to 5 over 95 trials). Secondly, formant data was smoothed by a robust version of local regression using weighted linear least squares fitting and a 1st degree polynomial model (span of the moving average = 5). This robust version assigns lower weight to outliers in the regression and zero weight to data outside six mean absolute deviations. Finally, formant values recorded from participant's pronunciations were averaged for three different time frames: Baseline trials (trials 6–20), Hold trials (trials 61–80), and End trials (trials 81–100).

Adaptive changes in formant frequency were calculated as the scalar projection of formant change in the direction opposite to the alteration, measured using the following equation (see also Niziolek and Guenther (2013), **Figure 2A**):

$$\begin{array}{l} \text{C}=\;\frac{\left(\begin{array}{c} \text{F}1_{\text{x}}-\text{F}1_{\text{b}}\\ \text{F}2_{\text{x}}-\text{F}2_{\text{b}} \end{array}\right)•\left(\begin{array}{c} 150\\ -300 \end{array}\right)}{335.41} \end{array}$$

where *F*1_*x*_ and *F*2_*x*_ are the formant values of production x, and *F*1_*b*_ and *F*2_*b*_ are the formant values of the average baseline production. The vector (150 300) is the inverse of the alteration, representing perfect adaptation, and the denominator 335.41 is the magnitude of the alteration in Hz (√[150^2^+300^2^]).

This equation computes the scalar projection of the difference vector (i.e., how a given trial's F1 and F2 values differed from those of the mean baseline production) onto the alteration vector (i.e., how much the feedback was shifted: −150 Hz in F1 and 300 Hz in F2).

*Adaptation* was defined as adaptive changes to formant frequencies measured at the end of the Hold phase, i.e., trials 61–80. To get a single measure of adaptation per subject, we used the median value from these trials.

*Aftereffect* was defined as adaptive changes to formant frequencies measured during the End phase, i.e., trials 81–100. To get a single measure of aftereffect per subject, we used the median value from these trials.

### Executive control tasks

Since we hypothesized that poor adapters might have poor domain-general conflict monitoring skills, and so would adapt less for the alteration, we tested participants individually on their executive control skills. Classical psychological measures of executive control include tasks where participants face conflicting information and have to resolve such conflict in order to correctly perform the task. The most widely used psychological measures of executive control are the Simon (Simon and Rudell, 1967), Flanker (Eriksen and Eriksen, 1974), and numerical Stroop (Tzelgov et al., 1992) tasks. Each of the three tasks involves a slightly different type of conflict: the conflict in the numerical Stroop task concerns abstract (numerical) values, the conflict in the Simon task relies on the irrelevant spatial information of the stimulus, and the conflict in the Flanker task comes from surrounding distracters (Paap and Greenberg, 2013). In order to test executive control skills broadly, participants were tested in each of the three tasks.

#### Numerical stroop task

In the numerical Stroop paradigm (Tzelgov et al., 1992; see also Duñabeitia et al., 2014; Antón et al., 2016), participants have to decide which of two digits simultaneously displayed on a screen is physically larger than the other. A correct execution of the task requires ignoring the numerical values of the digits that could facilitate (congruent trials) or interfere (incongruent trials) with the task. In congruent trials, the physical size of the digits and their numerical magnitude align (e.g., physically larger digits correspond to those with greater numerical values). In neutral trials, both digits have the same numerical value, and there is no matching or mismatching information about the numerical magnitude that could modulate the response. In incongruent trials, the physical magnitude of the digits and their location in the mental number line provide mismatching pieces of information (e.g., a big 3 and a small 7).

Forty-eight pairs of digits were presented, with one digit on the left and one digit on the right part of the screen. All digits were displayed in Courier New black font on a white background, small digits in size 32 and large digits in size 48. Sixteen pairs were congruent, 16 were incongruent, and 16 were neutral. Participants had to decide as fast and accurately as possible which digit was physically larger, pressing the left button of the keyboard when choosing the digit on the left, and the right button when choosing the digit on the right.

#### Flanker task

In the Flanker task (Eriksen and Eriksen, 1974), participants have to determine the direction of the arrow displayed at the center of the computer screen. A correct execution of the task requires ignoring the direction of the surrounding arrows (distracters). Distracters are 4 arrows (2 immediately to the left and 2 to the right of the central target arrow) that can facilitate (congruent trials) or interfere (incongruent trials) with the task. In congruent trials, the direction of distracters is identical to the direction of the target arrow. In neutral trials, distracters are horizontal lines with no left or right directionality. In incongruent trials, the direction of distracters is opposite to the direction of the target central arrow.

Forty-eight trials (sets of 5 arrows) were displayed at the center of the computer screen. All symbols were displayed in Courier New black font (size 48) on a white background (a dash mark for horizontal lines; the “less than” symbol for left arrow; the “greater than” symbol for right arrow). 16 trials were congruent, 16 were incongruent, and 16 were neutral. Participants had to decide as fast and accurately as possible which direction the central arrow pointed, pressing the left button of the keyboard when the arrow was pointing to the left, and the right button when the arrow was pointing to the right.

#### Simon task

In the Simon task (Simon and Rudell, 1967), participants have to press a left button when a square is displayed on the screen and a right button when a circle is displayed. A correct execution of the task requires ignoring the spatial location of the shape that could facilitate (congruent trials) or interfere (incongruent trials) with the task. In congruent trials, the shape requiring a left button press is displayed on the left of screen and vice versa. In neutral trials, the shapes are displayed at the center of the screen. In incongruent trials, the shape requiring a left button press is displayed on the right of the screen and vice versa.

Forty-eight trials (shapes) were presented one by one on the computer screen. Sixteen trials were congruent, 16 were incongruent, and 16 were neutral. Participants had to decide as fast and accurately as possible whether a circle or a square was displayed. Button press mappings were counterbalanced across participants.

#### Executive control tasks processing

The processing procedure was similar for the three executive control tasks. Incorrect responses (<2% of the data) and reaction times below or above 2.5 standard deviations from the mean in each condition for each participant (<2.5% of the data) were excluded from the latency analysis. For each task, cognitive control skills were measured as the difference in mean reaction time between congruent and incongruent trials (hereafter called “interference effect”), and then submitted to regression analyses. Given the very high accuracy in each participant and each task, accuracy scores were not included in the correlation analyses. For the sake of completeness, two other measures were calculated: The congruency effect was measured as the difference in mean reaction time between neutral and congruent trials. The incongruity effect was measured as the difference in mean reaction time between incongruent and neutral trials. Results were similar when submitting those measures to regression analyses.

It could be argued that auditory executive control tasks would have been better suited than visual ones. However, these tasks are claimed to reflect general executive control capacities in a modality-independent way (see for instance Roberts et al., 2006; Spagna et al., 2015). Moreover, it should be kept in mind that one of the aims of the current study was to tease apart effects attributable to executive control and those due to auditory acuity. For those two reasons, visual tasks were better suited for our study.

### Auditory acuity tasks

Four different auditory acuity tasks were used in this experiment (taken from Foster and Zatorre, 2010a,b; Voss and Zatorre, 2011). Two of them measured low-level auditory processes (pitch and loudness discrimination), and the other two evaluated higher-level auditory processing (simple melody discrimination and transposed melody discrimination). The two low-level tasks contained four blocks each while the high-level tasks consisted of two blocks each. Task order was counterbalanced.

#### Pitch discrimination task

Participants had to decide which of 2 tones was higher in pitch (ISI = 1,500 ms). They had to press the left mouse-button when the first sound was higher in pitch, and the right button when the second sound was higher. The reference tone was a 500 Hz pure tone (500 ms), and the task followed a 2-down/1-up staircase procedure (Levitt, 1970; the initial difference between the 2 tones was stepped down after two sequential correct responses and stepped up after a single incorrect response). This procedure produces runs of increasing and decreasing the difference between stimuli whose endpoints (reversal points) bracket the 71% discrimination threshold. The initial difference in frequency was 7%, and the initial step factor was 2 (to converge rapidly onto the subject's approximate threshold). After two reversals, the step factor was reduced to 1.25 to determine the threshold with greater precision. One staircase run was completed after 15 reversals, and the geometric mean of the value of the last eight reversals was taken as the threshold. The threshold was therefore unaffected by the choice of starting difference because the first 7 endpoints were not entered into the calculation. Four separate runs were conducted for each subject and averaged to produce the final discrimination threshold. The median of the geometric mean obtained for each of the four blocks was calculated and included in the final regression analysis.

#### Loudness discrimination task

Participants had to decide which of 2 tones was louder (ISI = 1,500 ms). The procedure was identical to that of the pitch discrimination task except that the tones differed in loudness, not pitch. The standard loudness reference of the tones was set at 65 dB sound pressure level and the initial difference was set at 10 dB. Participants had to press the left mouse-button when the first sound was louder, and the right button when the second sound was louder. As in the pitch discrimination task, the median of the geometric mean obtained for each of the four blocks was calculated and included in the final regression analysis.

#### High-level auditory discrimination tasks

For the two higher-level auditory tasks, subjects had to decide whether two successive sequences were identical or not via a button press on a 2-button mouse. The sequences were composed of 5–13 notes, and each note was 320 ms in duration, equivalent to eighth notes at a tempo of 93.75 beats per min.

In the *simple melody discrimination task*, participants had to decide whether 2 sequential melodies were identical or different. All stimuli were unfamiliar melodies in the Western major scale. In half of the trials (“different” trials), the pitch of a single note was changed, anywhere in the melody, by up to ±5 semitones (median of 2 semitones). The change maintained the key of the melody as well as the melodic contour. The number of notes in a melody was progressively increased during the task. The task was divided into two blocks.

The *transposed melody discrimination task* was identical to the previous task with 2 exceptions. First, all the notes of the second stimulus pattern were transposed 4 semitones higher in pitch (both in the “same” and “different” trials). Second, in “different” trials, one note was altered by 1 semitone to a pitch outside the pattern's new key, maintaining the melodic contour. This task therefore required the listener to compare the pattern of pitch intervals (frequency ratios) between each successive tone, and not the absolute pitches of each tone, since those were always different in the 2 melodies of the pair due to the transposition. The number of notes in a trial was progressively increased during the task. This task required a more abstract relational processing, as opposed to the simple melody task, which could be accomplished by direct comparison of the individual pitch values.

For the two high level auditory discrimination tasks, percentages of correct responses were calculated to be included in the regression analysis. Correct responses were “same” responses when the stimuli were identical and “different” responses when the stimuli were different. Total scores were divided by 120 (total number of trials).

## Results

### Auditory altered feedback

**Figure 1:**
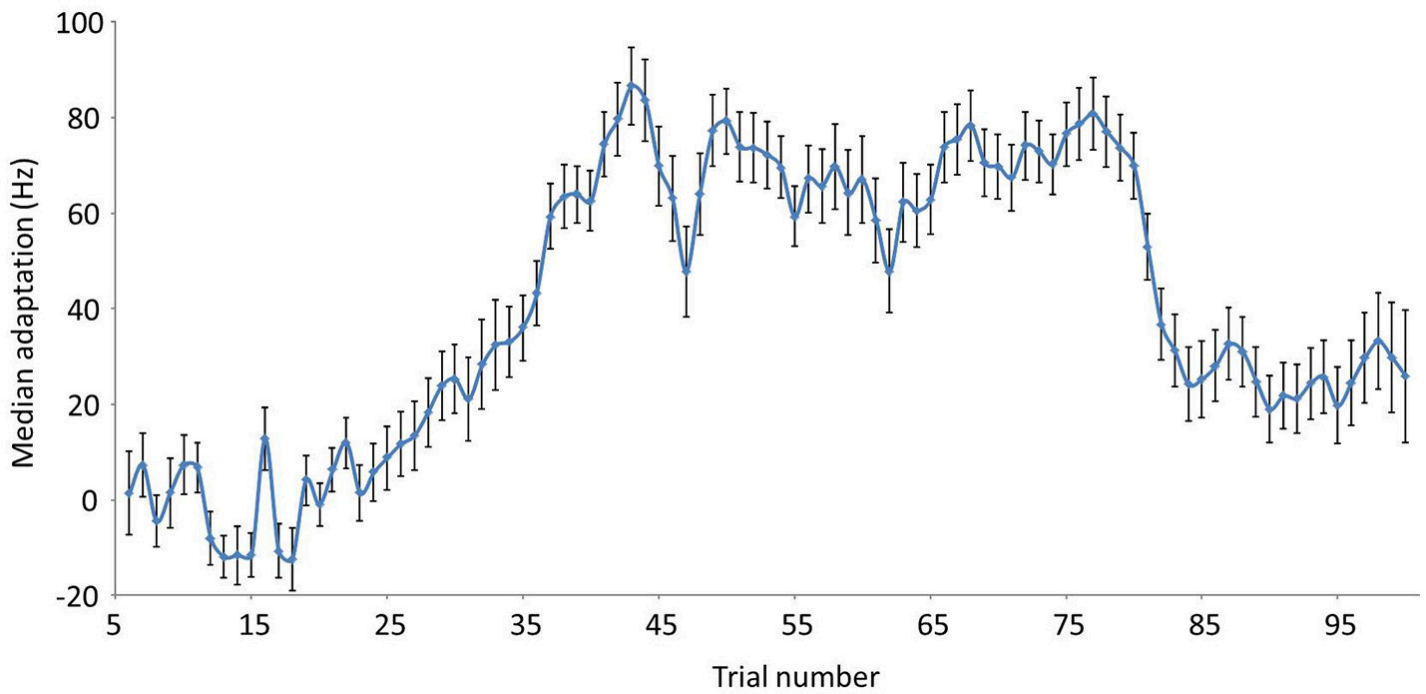
Median trial-by-trial adaptation and aftereffect values in Hz (for a 335.41 Hz maximal shift). Baseline trials = 5–20; Ramp trials = 21–40; Hold trials = 41–80; End trials = 81–100. Error bars reflect standard errors.

Median trial-by-trial adaptation for the 31 participants is shown in Figure 1. As a group, participants adapted progressively during ramp trials and reached a plateau during hold trials (41–80). Adaptation progressively decreased after abrupt removal of alteration (trials 81–100).

**Figure 2:**
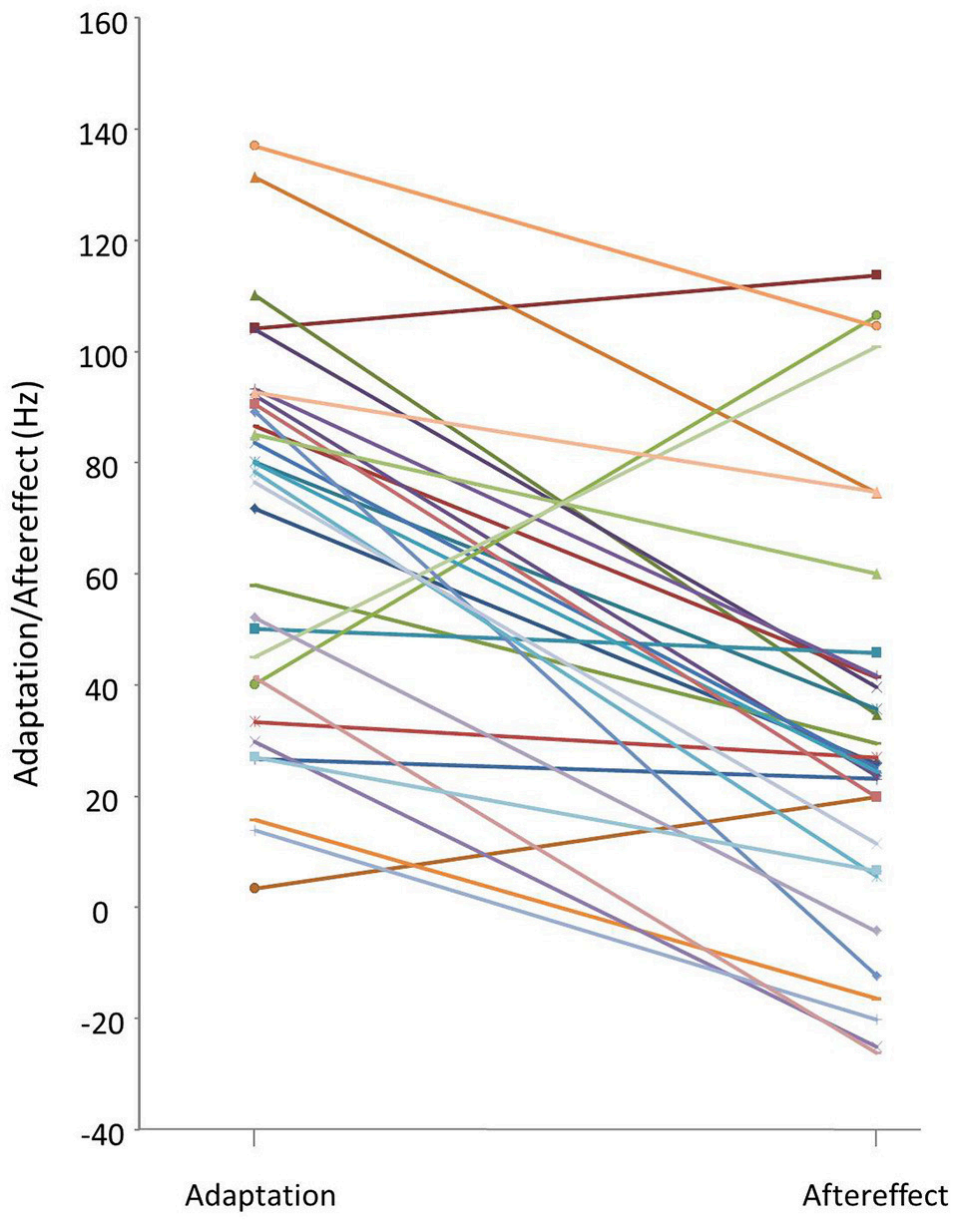
Individual median adaptation and aftereffect values in Hz (for a 335.41 Hz maximal shift). Adaptation = Median shift in production in trials 61–80. Aftereffect = Median shift in production in trials 81–100. Each line links online adaptation and aftereffect values for one participant.

Median adaptation and aftereffect values obtained for each participant in the altered auditory feedback paradigm are shown in Figure 2. The majority of the participants adapted during full alteration (hold trials) but a large individual variability was observed. Adaptation values ranged from 4 to 137 Hz (median = 78 ± 35), aftereffect values from −26 to 114 Hz (median = 26 ± 39). Note that the magnitude of the shift was the same for all participants (335.41 Hz), meaning that adaptation ranged from 1.0 to 40.8% (median = 23.3) of maximal alteration, aftereffect ranged from −7.8 to 33.9% (median = 7.7).

### Executive control

The interference effects obtained in the three executive control tasks also varied across participants. Interference effects ranged from −120 to 30 ms (median = −46 ± 37) in the Flanker task, from −123 to 4 ms (median = −40 ± 31) in the Simon task and from −117 to 27 ms (median = −20 ± 37) in the Stroop task (see Appendix A for individual values).

### Auditory acuity

Median discrimination thresholds ranged from 0.2 to 3.1 (median = 1.2 ± 0.8) for pitch and from 0.3 to 2.1 (median = 0.8 ± 0.4) for loudness. Percentages of correct responses in the melody discrimination tasks ranged from 67 to 90% (median = 77 ± 0.6; see Appendix A).

### Regression analyses

Regression analyses were performed on the data across subjects, comparing adaptation and aftereffect to the series of measures obtained in auditory acuity and executive control tasks.

We first examined correlations between the following variables: (1) Interference effects in the three executive control tasks (numerical Stroop, Simon and Flanker); (2) Loudness and Pitch discrimination thresholds; (3) Percentages of correct responses (%CR) in the Simple Melody and Transposed Melody discrimination tasks.

Not surprisingly, and as it has been already shown in earlier studies (e.g., Duñabeitia et al., 2014), the three interference effects were not significantly correlated with each other (all *p* > 0.71), suggesting that in spite of the similarities between these tasks, the cross-task consistency of the indices is markedly low (see also Miyake and Friedman, 2012). Thus, regression analyses were performed using the three interference scores.

Loudness and Pitch discrimination thresholds were not significantly correlated with each other (*p* = 0.68), so both values were entered in regression analyses.

Percentages of correct responses in the Simple and Transposed melody tasks were significantly correlated (*R*^2^ = 0.38, *p* = 0.035). Thus, a combined score was measured, being the percentage of correct responses in both the tasks.

In sum, the regression analyses were performed comparing adaptation and aftereffect to (1) Interference effects in the three executive control tasks (numerical Stroop, Simon and Flanker), (2) Loudness and Pitch discrimination thresholds, (3) Percentages of correct responses in the Melody discrimination tasks (combined scores of Simple and Transposed). The 6 measures entered in regression analyses were not significantly correlated (all *p* > 0.26).

#### Regression analysis on median adaptation

Automatic backward multiple regression was used to assess the associations between median adaptation and the six independent variables listed above. The six independent variables were entered in the first model, and variables irrelevant for the model (*p* > 0.10) were removed step-by-step until the model including the more relevant (significant) variables was obtained. The initial model had an adjusted *R*^2^ of 0.61 [Standard error of the estimate = 24.2; *F*_(6, 24)_ = 6.18, *p* = 0.001]. The final model obtained had an adjusted *R*^2^ of 0.55 [Standard error of the estimate = 24.5; *F*_(3, 27)_ = 10.98, *p* < 0.001].

Standardized Beta Weights, *t*, and *p*-values for each variable entered into the initial model are reported in Table 1, as well as values for the remaining variables in the final model.

**Table 1:** Backward multiple Regression of median adaptation showing Standardized Beta Weights, *t*, and *p*-values for each variable entered into the initial model (upper panel) and into the final model (lower panel).

**Predictor variables**	**Beta weights**	***t*-values**	***p*-values**
**INITIAL MODEL (6 PREDICTORS)**			
Interference Flanker	−0.21	−1.36	0.19
Interference Simon	0.024	0.18	0.86
Interference Stroop	−0.20	−1.37	0.19
Loudness threshold	−0.45	−2.90	0.008
Pitch threshold	−0.33	−2.22	0.036
Melody discrimination %CR	0.39	2.74	0.011
**FINAL MODEL (3 PREDICTORS)**			
Loudness threshold	−0.31	−2.28	0.031
Pitch threshold	−0.37	−2.88	0.008
Melody discrimination %CR	0.45	3.34	0.002

*%CR refers to the percentages of correct responses in the Melody discrimination tasks*.

The regression model revealed that melody discrimination score, loudness threshold and pitch threshold were significant and independent predictors of adaptation. Overall, the model explained 55% of the variance in adaptation. Cognitive function skills were not significant predictors of adaptation. Note that using mean instead of median individual values did not change the outcome of the models (see Appendix A for individual values; median and mean adaptation scores highly correlated, *r* = 0.987).

Those results were confirmed by independent correlation analyses. Median adaptation was significantly correlated with Loudness threshold (*r* = −0.48, *p* = 0.006), Pitch threshold (*r* = −0.40, *p* = 0.027) and Melody discrimination score (*r* = 0.55, *p* = 0.001). Median adaptation was not correlated to interference effects in the Flanker task (*r* = −0.11, *p* = 0.55), Simon task (*r* = 0.20, *p* = 0.28), or Stroop task (r = −0.08, *p* = 0.68).

The better auditory acuity is (larger melody discrimination scores and smaller loudness and pitch discrimination threshold), the larger adaptation to alteration.

#### Regression analysis on median aftereffect

Automatic backward multiple regression was used to assess the associations between median aftereffect and the six independent variables listed above. A seventh variable was added, which was median adaptation. The seven predictors were entered in the first model, and variables irrelevant for the model (*p* > 0.10) were removed step-by-step until the model including the more relevant (significant) variables was obtained. The initial model had an adjusted *R*^2^ of 0.30 [Standard error of the estimate = 36.7; *F*_(7, 23)_ = 1.42, *p* = 0.25). The final model obtained had an adjusted *R*^2^ of 0.23 [Standard error of the estimate = 34.4; *F*_(1, 29)_ = 8.59, *p* = 0.007).

Standardized Beta Weights, *t*, and *p*-values for each variable entered into the initial model are reported in Table 2, as well as values for the remaining variables in the final model.

**Table 2:** Backward multiple Regression of median aftereffect showing Standardized Beta Weights, *t*, and *p*-values for each variable entered into the initial model (upper panel) and into the final model (lower panel).

**Predictor variables**	**Beta weights**	***t*-values**	***p*-values**
**INITIAL MODEL (7 PREDICTORS)**			
Interference Flanker	0.21	0.96	0.35
Interference Simon	0.08	0.44	0.66
Interference Stroop	−0.02	−0.11	0.92
Loudness threshold	−0.11	−0.46	0.65
Pitch threshold	−0.05	−0.21	0.84
Melody discrimination %CR	−0.16	−0.72	0.48
Median adaptation	0.50	1.79	0.09
**FINAL MODEL (1 PREDICTOR)**			
Median adaptation	0.48	2.93	0.007

*%CR refers to the percentages of correct responses in the Melody discrimination tasks*.

Overall, the regression model explained only 23% of the variance in aftereffect, and it revealed only one significant predictor of aftereffect, which is adaptation. The relation between median adaptation and median aftereffect was confirmed by an independent correlation analysis, showing a large correlation between the two variables (*r* = 0.48, *p* = 0.007). Note that using mean instead of median individual values did not change the outcome of the models (see Appendix A for individual values; median and mean aftereffect scores highly correlated, *r* = 0.995).

## Discussion

In the present study, we explored the influence of general executive control skills, and auditory acuity on altered feedback control. Our main hypothesis was that those cognitive skills would influence auditory feedback control and explain the large variability usually observed in auditory altered feedback paradigms. First, we observed a large individual variability in adaptation and aftereffect, as previously reported in several similar studies (Houde and Jordan, 2002; Purcell and Munhall, 2006b; Villacorta et al., 2007; Cai et al., 2010). Second, the main outcome of the study is that auditory acuity explains a considerable amount of variability in adaptation to altered feedback. On the other hand, general executive control does not seem to play a significant role in adaptation and aftereffect.

### Adaptation to altered feedback and executive control

The influence of general executive control skills on adaptation to altered feedback was explored in the present study because of one tentative explanation for variability in adaptation: poor adapters might be poor in conflict monitoring and thus poor in resolving conflict emerging from feedback alteration. The present study did not reveal any major influence of cognitive control skills in the way participants adapted to the feedback alteration.

As far as we know, the relationship between executive control resources and adaptation was explored in one previous study. Testing the assumption that processing of altered auditory feedback (conflict detection) involves executive control, Pfordresher and Beasley (2014) had participants perform an altered auditory feedback task while simultaneously performing a secondary executive control task. The primary task was to perform piano melodies on a keyboard while hearing, in the critical condition, auditory feedback altered in pitch on some trials. Their hypothesis was that the secondary executive control task should reduce the disruptive effect of altered auditory feedback, if the primary task (monitoring of auditory feedback) indeed involved executive functioning. They observed that the interplay between feedforward and feedback information in motor control was not influenced by the availability of executive resources (Pfordresher and Beasley, 2014). Thus, their results did not confirm any involvement of executive control in auditory feedback processing suggested by cognitive control models (e.g., Botvinick et al., 2001; Holroyd et al., 2005). The authors interpreted their results based on the framework of motor-control models (internal forward models presented in introduction) that do not make claims about implication of executive resources in speech motor control (e.g., Hickok et al., 2011; Houde and Nagarajan, 2011; Tourville and Guenther, 2011).

The results of our experiment are consistent with this interpretation, as we did not observe any direct link between general executive control and adaptation/aftereffect to alteration. Note that executive control skills were not correlated either with adaptation when considering only participants with the highest auditory acuity. Thus, it seems that the primary role of auditory acuity in feedback perception was not a limiting factor for the influence of executive control resources. Altogether, those results suggest, in line with previous studies, that auditory feedback control during self-produced speech would be independent of general executive function skills (Hu et al., 2015). Thus, the emerging picture seems to be that auditory feedback processing does not depend on general cognitive control skills, but may involve attentional resources. In fact, several previous experiments showed that attention plays a critical role in adaptation (see for instance Tumber et al., 2014; Liu et al., 2015; Scheerer et al., 2016).

One caution should be taken though regarding the null effect obtained here. Despite the claim that they reflect domain-general executive control capacities, the tasks used in the present study specifically test conflict resolution between two sources of information, one being task-relevant and the other one being distracting. Two major differences between the type of control operating in speech production and executive control tasks could account for the absence of correlation between adaptation and executive control skills: (1) In speech production, several sources of information (and not only two) have to be considered in conflict resolution (e.g., feedforward, auditory feedback and somatosensory feedback). (2) In speech production, various sources of information are presumably task-relevant (with no clear distinction between task-relevant and distracting information as in executive control tasks). Consequently, we can conclude that domain-general executive control capacities as they are tested through the classical Simon, Flanker and Stroop tasks (Roberts et al., 2006; Spagna et al., 2015) do not explain adaptive behavior to feedback alteration. Further studies should explore precisely the link between linguistic and non-linguistic conflict resolution when several sources of task-relevant information are at play, as it is the case in speech production.

Here, we suggest that domain-general cognitive control skills do not play a relevant role in adaptation to alteration of formant frequencies. Note that this conclusion might be restricted to formant frequency manipulations given that previous studies showed attentional effects in adaptation to pitch alteration. It might be that general cognitive control skills also play a relevant role in adaptation to pitch alteration, given that differential mechanisms underlie the processing of fundamental and formant frequencies (Mollaei et al., 2016). Note also that our conclusion is restricted to the manipulation we included, being that feedback alteration was progressive, subtle, and not consciously perceived by the participants (none of them reported at the end of the experiment having perceived the alteration during ramp and hold trials). Further studies should investigate the role of cognitive control in adaptation, using abrupt, consciously perceived alterations. Cognitive control skills may play a significant role when consciously perceived alterations can be ignored (inhibited). In this context, higher cognitive control skills, especially inhibitory capacities, might be associated to lower adaptation.

### Adaptation to altered feedback and auditory acuity

The influence of auditory acuity on adaptation to altered feedback was explored in the present study because of one of the main predictions of the DIVA model: Participants with higher auditory acuity were expected to better detect feedback that falls farther from their vowel target regions and thus to better adapt their speech production to alterations in their auditory feedback. We confirmed this prediction by showing that the better the auditory acuity of the participants, the larger the amount of adaptation to alteration. Participants with lower pitch and loudness discrimination thresholds, and participants with better accuracy in melody discrimination, adapted more to the altered feedback. Those results are in line with Villacorta and colleagues' observation that auditory acuity positively correlates with adaptation (Villacorta et al., 2007). Interestingly, our results go beyond this previous study, by showing that general auditory acuity partially explains adaptation, and not only auditory acuity in the same dimension as the one altered in feedback (F1 dimension in Villacorta et al., 2007). Here, we showed that adaptation in F1/F2 is significantly correlated with dimensions not manipulated in the core experiment (pitch, loudness, and melody discrimination). Thus, we can conclude that adaptation to altered auditory feedback is very well-predicted by general auditory acuity, as suggested by the DIVA model (Guenther et al., 1998, 2006; Tourville and Guenther, 2011). We provided evidence that participants with higher auditory acuity are better in adapting their production until formant values of their auditory feedback falls into the target sound region. An important question remains open, which is what should be considered the “target region.” Previous evidence suggests that the target region does not encompass all the variability observed in phoneme production, even within an individual, since adaptation to altered feedback take place even within a phoneme boundary (Niziolek and Guenther, 2013). Furthermore, besides a large individual variability in adaptation, most previous studies (as well as the present one) reveal that participants never fully compensate for the feedback alteration. This phenomenon could be explained in several ways: (1) “target regions” to be reached during production might be large enough so that a certain amount of discrepancies between production and feedback perception would still fall into this region and not be considered an error. (2) Alternatively, some variability in production might be expected and tolerated by the internal model, so that discrepancies that do not exceed a certain threshold would not invoke correction (Purcell and Munhall, 2006b). (3) Additionally, complete adaptation would entail a large mismatch in somatosensory feedback which is not tolerated by the internal model. Further investigation is needed to fully describe those corrective mechanisms and precisely define what should be considered a “target region” to be reached in speech production.

### Alternative explanations for variability in adaptation

Another potential explanation for variability in adaptation put forward by Houde and Jordan (2002) would be that poor adapters in production are indeed good adapters in perception -that is, that these subjects accept the altered feedback as an adequate example of the vowel category they are attempting to produce (see Shiller et al., 2010). In fact, it has been shown that speech perception as well as speech production can exhibit adaptation (Cooper, 1979) and previous studies nicely showed a great negative correlation between susceptibility to somatosensory feedback alterations and auditory feedback alterations (Lametti et al., 2012). In the present study, we observed that poor adapters in production were the ones with lower auditory acuity. Thus, based on Houde and Jordan's framework, it seems that being a good adapter in perception does not mean having high auditory acuity. No conclusion can be drawn on this topic since we did not test perceptual adaptation in the present study. Thus, further research should investigate the interplay between adaptation in production, adaptation in perception, and auditory acuity.

Another alternative explanation for variability in adaptation not tested here is that poor adapters would have a weak auditory-motor link (suboptimal internal model). Pfordresher and Beasley (2014), for instance, proposed that poor singers suffer from vague or distorted predictions (made by the forward model), which would make them “deaf” to subtle alterations in auditory feedback. The present results cannot shed light on this hypothesis that should be tested in further studies.

It is important to note that we did not observe clear correlations between auditory acuity (or cognitive control skills) and aftereffect to altered auditory feedback. Thus, it seems that the cognitive functions at play during adaptation and aftereffect are different, at least partially. While adaptation is significantly driven by auditory acuity, it seems that aftereffects are less related. In other words, our results suggest that auditory acuity influences online rather than long-term adaptation of motor commands. Individual adaptation and aftereffect values were highly positively correlated (Pearson correlation = 0.48, *p* = 0.007), revealing that participants who adapted more to the altered feedback (the ones with better auditory acuity) were the most persistent ones (larger aftereffects). If auditory acuity was driving the aftereffects, participants with better auditory acuity should be the ones with production closer to baseline after removing alteration (smaller aftereffects). This result tends to confirm that persistence in adaptation (aftereffects) is likely driven by other factors. We tentatively propose that flexibility should be at play in production during the End phase, given that previous recalibration of the target sound has to be overcome in order to get production back to baseline. Thus, shifting skills (i.e., ability to display flexibility when facing task changes), measured through the Wisconsin Card Sorting Test for instance (Monchi et al., 2001), might be relevant predictors for aftereffects. Aftereffects are also likely to be linked to memory, since the more a sound was adapted, the more persistent aftereffects were observed in the End phase (see Jones and Munhall, 2005; Purcell and Munhall, 2006b for similar argument). Further studies will be needed to identify the main components explaining precisely variability not only in online adaptation to altered feedback, but also in long-term adaptive changes to the forward model.

### Influence of native language on adaptation

Finally, even if it was not the main aim of the present study, it is important to note that we replicated, in Spanish native speakers, adaptation effects previously observed mainly in English native speakers. Thus, speakers of a language with a sparse vocal space (Spanish: 5 vowels) adapt to alterations in auditory feedback similarly to speakers of a dense vocal space language in which vowels overlap (English: 13–15 vowels).

Previous research on adaptation to altered feedback has mainly examined English vowels (with a few exceptions like Mandarin vowels, e.g., Cai et al., 2010). Averaged adaptation has generally been reported to be around 30% of maximal alteration. For instance, Villacorta et al. (2007) observed that North American English speakers had a mean adaptation of 35 and 50% for a shift-down and shift-up of F1, respectively. Purcell and Munhall (2006b) observed that Canadian English speakers had a mean adaptation of 29 and 30% for shift-down and shift-up of F1, respectively (averaged of 28% in the 3 conditions of experiment 2). In an experiment more similar to ours since both F1 and F2 were altered, MacDonald and colleagues reported a mean adaptation of 25–30% of the magnitude of the alteration (MacDonald et al., 2010). In the present study, mean adaptation was around 20% of maximal perturbation. Thus, Spanish natives adapted to feedback alteration as English natives do, and in a similar ratio. We can argue that Spanish speakers adapt to alteration, but we cannot argue whether they adapt equally or less than English natives, since we do not have possible direct group comparison. Furthermore, factors other than vocal space density probably influence the amount of adaptation. For instance, MacDonald et al. (2010) showed that when larger alteration levels were applied to auditory feedback, adaptation reached a plateau and even decreased. Thus, further studies should directly compare Spanish and English natives in a similar auditory feedback alteration paradigm in order to investigate potential effects of vocal space density on the magnitude of adaptation. One cross-language formant perturbation study has explored this (Mitsuya et al., 2011). The authors compared adaptation to F1 alteration in English (dense vocal space) and Japanese native speakers (sparse vocal space of 5 vowels). They observed that English and Japanese participants uttering native vowels adapted equally when F1 was decreased. When F1 was increased, Japanese speakers did not adapt as much as English speakers (Mitsuya et al., 2011). Those results suggest that some level of phonological properties of the languages (such as vocal space density) might influence adaptation to feedback alteration.

## Author contributions

CM, CN, JD, MC, and JH developed the study concept and design. Testing, data collection, and data analyses were performed by CM, CN, JD, JH, AP, and DH. All authors contributed to data interpretation. CM drafted the manuscript and all other authors provided critical revisions. All authors approved the final version of the manuscript for submission.

### Conflict of interest statement

The authors declare that the research was conducted in the absence of any commercial or financial relationships that could be construed as a potential conflict of interest.

## Footnotes

^1^Note that other factors are known to influence which sounds are considered errors in production. The lexical status of the production, for instance, influences adaptation to alteration, participants tending to avoid crossing word/non-word boundaries (see for instance Bourguignon et al., 2014). Such influence of lexicality was avoided in the present study, since all sounds being produced and/or perceived were Spanish non-words (i.e., /pep/, /pip/, /pap/; see Method section).
