# Supplementary material for: Online Adaptation to Altered Auditory Feedback Is Predicted by Auditory Acuity and Not by Domain-General Executive Control Resources
Source: Front Hum Neurosci. 2018 Mar 12;12:91. doi: 10.3389/fnhum.2018.00091 (PMC5857594; doi:10.3389/fnhum.2018.00091)
Supplement: Supplementary file 1 [file Table1.DOCX]

**Appendix A**

*Table A1:* Individual data (31 participants in rows) for interference effects in the Flanker (**Interf Flanker**), Simon (**Interf Simon**) ans Stroop (**Interf Stroop**) tasks, for loudness (**Loudness Thresh**), pitch (**Pitch Thresh**) and melody (**Melody Discr**) discrimination thresholds, and for median adaptation (**Med Adapt**), median aftereffect adaptation (**Med Aft**), mean adaptation (**Mean Adapt**) and mean aftereffect adaptation (**Mean Aft**), used in regression analyses.

| Interf Flanker | Interf Simon | Interf Stroop | Loudness Thresh | Pitch Thresh | Melody Discr | Med Adapt | Med Aft | Mean Adapt | Mean Aft |
| --- | --- | --- | --- | --- | --- | --- | --- | --- | --- |
| -32.0 | -40.7 | 20.6 | 0.63 | 0.60 | 0.83 | 72 | 26 | 71 | 30 |
| 17.0 | -14.5 | -59.9 | 0.38 | 2.45 | 0.85 | 104 | 114 | 101 | 121 |
| -51.6 | -23.6 | -66.8 | 0.44 | 2.98 | 0.85 | 110 | 35 | 112 | 38 |
| -113.9 | -44.1 | -3.4 | 0.78 | 0.62 | 0.90 | 104 | 40 | 101 | 41 |
| -55.6 | -27.7 | -30.4 | 0.76 | 1.22 | 0.79 | 80 | 36 | 75 | 34 |
| -47.4 | -29.6 | -66.1 | 0.87 | 2.20 | 0.70 | 4 | 20 | 2 | 21 |
| -27.9 | -48.5 | -22.6 | 1.25 | 3.13 | 0.70 | 27 | 23 | 23 | 20 |
| -119.5 | 4.1 | -55.4 | 1.25 | 0.82 | 0.78 | 87 | 42 | 83 | 43 |
| -45.8 | -10.1 | 7.1 | 0.81 | 2.47 | 0.75 | 58 | 30 | 59 | 27 |
| -114.2 | -43.2 | -66.8 | 0.51 | 0.41 | 0.73 | 92 | 24 | 93 | 24 |
| 5.1 | -41.8 | -62.1 | 0.36 | 0.98 | 0.73 | 50 | 46 | 58 | 49 |
| -32.9 | -11.7 | -60.5 | 1.12 | 0.23 | 0.76 | 131 | 75 | 131 | 70 |
| -68.8 | -29.7 | -4.8 | 0.55 | 0.78 | 0.77 | 84 | 25 | 82 | 26 |
| -37.5 | -119.3 | -6.5 | 0.83 | 2.78 | 0.68 | 33 | 27 | 28 | 27 |
| -52.1 | -43.3 | -15.7 | 1.01 | 0.87 | 0.75 | 40 | 106 | 35 | 119 |
| -55.0 | -21.2 | 12.1 | 0.35 | 1.12 | 0.78 | 93 | 42 | 100 | 44 |
| -38.0 | -35.8 | -9.3 | 0.58 | 1.20 | 0.84 | 80 | 24 | 66 | 27 |
| -72.5 | -0.6 | -4.5 | 1.54 | 1.55 | 0.76 | 16 | -16 | 18 | -15 |
| 0.8 | -39.9 | -30.3 | 0.93 | 0.71 | 0.80 | 89 | -12 | 91 | -16 |
| -36.7 | -83.2 | -19.9 | 0.32 | 1.93 | 0.79 | 90 | 20 | 93 | 17 |
| -39.4 | -19.4 | 27.2 | 0.44 | 0.79 | 0.78 | 85 | 60 | 84 | 64 |
| -53.0 | -28.7 | 19.6 | 1.03 | 1.19 | 0.72 | 30 | -25 | 29 | -23 |
| -67.0 | -67.2 | -14.8 | 0.34 | 1.03 | 0.79 | 78 | 6 | 78 | 7 |
| -57.7 | -48.4 | -85.6 | 0.53 | 0.84 | 0.84 | 137 | 105 | 137 | 100 |
| -17.8 | -35.9 | -33.8 | 1.07 | 1.68 | 0.75 | 14 | -20 | 8 | -28 |
| 30.1 | -49.6 | -116.6 | 0.70 | 2.94 | 0.87 | 41 | -26 | 18 | -29 |
| 10.4 | -74.8 | 8.2 | 0.38 | 1.48 | 0.78 | 45 | 101 | 48 | 109 |
| -93.2 | -60.4 | 0.0 | 0.97 | 0.82 | 0.67 | 52 | -4 | 53 | -4 |
| -93.7 | -84.9 | -95.6 | 2.15 | 1.43 | 0.73 | 27 | 7 | 31 | 14 |
| -18.8 | -0.6 | 9.7 | 0.28 | 0.33 | 0.71 | 93 | 75 | 89 | 69 |
| -39.1 | -122.8 | -28.4 | 0.97 | 1.82 | 0.76 | 76 | 12 | 76 | 13 |
